# Scheduling Sensor Duty Cycling Based on Event Detection Using Bi-Directional Long Short-Term Memory and Reinforcement Learning

**DOI:** 10.3390/s20195498

**Published:** 2020-09-25

**Authors:** Muhammad Diyan, Murad Khan, Bhagya Nathali Silva, Kijun Han

**Affiliations:** 1 School of Computer Science and Engineering, Kyungpook National University, Daegu 41566, Korea; m.diyan@knu.ac.kr (M.D.); mkhan@knu.ac.kr (M.K.); 2 Department of Computer Engineering, Faculty of Engineering, University of Sri Jayewardenepura, Nugegoda 10250, Sri Lanka; nathalis@netopia.knu.ac.kr

**Keywords:** smart homes, event detection, activity detection, deep learning, long-short term memory, sensor duty cycling

## Abstract

A smart home provides a facilitated environment for the detection of human activity with appropriate Deep Learning algorithms to manipulate data collected from numerous sensors attached to various smart things in a smart home environment. Human activities comprise expected and unexpected behavior events; therefore, detecting these events consisting of mutual dependent activities poses a key challenge in the activities detection paradigm. Besides, the battery-powered sensor ubiquitously and extensively monitors activities, disputes, and sensor energy depletion. Therefore, to address these challenges, we propose an Energy and Event Aware-Sensor Duty Cycling scheme. The proposed model predicts the future expected event using the Bi-Directional Long-Short Term Memory model and allocates Predictive Sensors to the predicted event. To detect the unexpected events, the proposed model localizes a Monitor Sensor within a cluster of Hibernate Sensors using the Jaccard Similarity Index. Finally, we optimize the performance of our proposed scheme by employing the Q-Learning algorithm to track the missed or undetected events. The simulation is executed against the conventional Machine Learning algorithms for the sensor duty cycle, scheduling to reduce the sensor energy consumption and improve the activity detection accuracy. The experimental evaluation of our proposed scheme shows significant improvement in activity detection accuracy from 94.12% to 96.12%. Besides, the effective rotation of the Monitor Sensor significantly improves the energy consumption of each sensor with the entire network lifetime.

## 1. Introduction

Wireless Sensor Network (WSN) is the key component in the Internet of Things (IoT) technology and plays a key role in people’s lives to enabling many smart services from smart living to energy saving. These smart services are hinged with the interconnectivity of large scale smart sensors, which continuously monitor human activities and environmental dynamics to collect data for various IoT applications [1,2,3]. It is mattered to enhance the living standard of the residents by adopting smart devices and ubiquitous sensors [4]. In an upgraded smart home, the entire smart devices are interconnected with WSN, which assesses the residents through comfort and independent automated smart services. These smart devices interact with the residents and learn through resident behavior and construct an interactive environment. For instance, the smart home application services can monitor the daily routine of the residents to give a convenient environment, e.g., control and manage temperature, light intensity, and humidity, etc., to improve the quality of resident’s life. According to [5], it is predicted that the number of smart devices connected to the internet will be three-fold than the global population. The growing number of IoT-enabled smart innovative services incorporate smart home applications, surveillance applications, and health care applications [6]. Most of these applications incorporate with irregular duty cycle, battery-powered sensors with no alternative energy resources. To avoid missing activities, these sensors perform their duty and keep active for a very long time period to gather the sensory data. Therefore, continuous and extensive use of battery-powered sensor drains their residual energy unexpectedly. An intelligent Sensor Duty Cycling (SDC) scheme is needed to design in a way to cover problems like irregular SDC, missing activities, energy depletion effectively and can sense all the activity, minimize the energy constraints, and maximize the sensor lifetime [1].

Systematic SDC is one of the key ideas for detecting the activities of a smart home, resulting in the development of a true smart home system. Since SDC is not a new topic, a number of studies have been proposed. However, some of these studies are restricted to use the WSN continuously and collect the data with no missing of useful activities [7]. Moreover, ambient wireless sensors, which incorporate light, temperature, door, and contact positioning sensors, are low-cost and battery- powered sensor. Energy depletion is the major challenge for these sensors due to extensive use and being active all the time in the smart home environment. In this case, the WSN needs to be operational all the time; some studies have addressed the problem by using SDC to detect activities precisely and consume less residual energy [8,9]. These schemes control the extensive and continuous usage of the sensor by employing sleep mode when not operational. The main challenge in the said schemes is it may miss the useful activities during the sleep mode. In many IoT applications, it may not be convenient. Therefore, an activity prediction-based SDC scheme is designed to tackle the accuracy and energy efficiency problem simultaneously [9]. In the prediction based schemes, the approaching activities are predicted, and against these activities, the cluster of sensors are activated. Here, the activity is mapped with the pattern of approaching events; for example, bathing activity incorporates the events to make water hot, access to sink, hairdryer, and curtain. Still, the predictive-based approach is not suitable for the real smart home environment. When an incident or daily routine of the resident slightly deviates from its normal, the resident performs unexpected activities, which can’t be predicted. For instance, if a resident prepares the dinner late than usual or falls in the stairs. However, when these activities are continuously avoided, the reliability of the smart home system will lead to degradation. To cope with the challenges present in the prediction scheme, event-based SDC is introduced [10]. This scheme selects a sentry sensor that awakes to monitor the resident activities of its interest, while the rest of the sensors are asleep. When the sentry sensor detects activity, the rest of the sensors awake and take part in event monitoring. Even so, the event-based sensor approach has two issues, which need to be resolved. First, the location of the sentry sensor should be selected in a way to improve the activity detection accuracy. Second, it is observed that the life of the sentry sensor in the event-based sensor approach will be less than the other sensor in the cluster due to the extensive use of sentry sensors while leading to WSN degradation.

Human Activity Detection (HAD) is one of the most widely used source keys of smart home and has been extensively used for real smart home automation and assisted living domains to provide convenient smart services for the human [11]. The main objective of the HAD is to locate and detect human daily routine activities and unexpected complex activities in real-time by processing ambient sensors data [4]. The accomplishment of this objective relies on the capabilities of the HAD system to learn and track human daily life routine activities [12]. However, the real-time dynamic environment is composite and unpredictable, and the collected sensory data may be noisy and ambiguous. Therefore, to address these challenges, some efforts based on Machine Learning (ML) have been done to uncover the data and learn meaningful information from the data to predict the human activities accurately. Most recent ML approaches are hinge with the static models. Besides, such approaches are unsatisfactory in front of the dynamic environment. However, these algorithms have their own advantages despite that none of them overcome other problems in different domains [7]. The rising attraction in Deep Learning (DL) model for HAD has shown significant improvement in activity detection via prediction. These models are trained with the sensory data and learn human behavior from the nonlinear raw data using a layered architecture. The popular DL techniques, i.e., Recurrent Neural network (RNN), Long Short-Term Memory (LSTM), Convolutional Neural Network (CNN), and Deep Neural network (DNN), are widely adopted to predict activity detections [13,14]. In particular, LSTM has been broadly adopted to uncover the HAD. LSTM is a unique model of RNN, which is a sub-category of the Artificial Neural Network where interconnections amid in cells establish a cycle and create a fully internally linked network. This RNN design results in a temporal response. RNN uses its internal memory to process various inputs, which exhibit the problem of memory exploding (memory vanishing) and gradient problem. The said issues in RNN are unable to address the long-term dependency issue and very challenging to train. Therefore, LSTM addresses the problem to overcome the issue with RNN by incorporating the gating technique and gives effective results than conventional RNN.

Unlike from the former approaches, in this research work, we propose Energy and Event Aware-Sensor Duty Cycling (EEA-SDC) model to detect a particular event and schedule sensor duty cycling, which improves the battery lifetime of the embedded ambient sensors of the smart home appliances in a Home Area Network (HAN). In a smart home environment, the residents interact with various smart appliances, resulting in two types of the event of activities, i.e., expected and unexpected events. These events are performed in a sequential time frame, from which the future can be forecasted by solving the sequential time frame forecasting problem. Therefore, we tackle the expected and unexpected events in a smart home environment via Predictive Sensors (PS) and Monitor Sensors (MS) accordingly. Moreover, the sensor residual energy consumption with the overall network lifetime is handled through the appropriate scheduling of PS and MS duty cycling. Within the Hibernate Sensors (HS), the MS is elected on the basis of three parameters (1) Location ID of the sensor, (2) Residual energy of the sensor, (3) Event detection frequency of the sensor in the previous time frame. However, the PS is activated against the predicted event from the available historical data with the Bi-Directional LSTM (BiLSTM) method. Despite that, our model is still missing some unexpected events. Hence, we optimize our model and boost the performance of the BiLSTM by adopting a Reinforcement Learning (RL) model-free method called Q-Learning. We model the problem of missed and undetected events as an RL problem and solve with the standard Q-Learning algorithm.

The rest of the paper is arranged as follows: Section 2 illustrates a comprehensive literature review. Section 3 explains the methodology of the proposed scheme. Further, the evaluation and analysis of the experimental results are explained in Section 4. Finally, the conclusion is summarized in Section 5.

## 2. Literature Review

### 2.1. Human Activity Detection (HAD) in Smart Home

The real smart home is incorporated by ambient sensors, which monitor the resident daily activities like sleeping, toileting, cooking, eating, washing, leave home, etc. Interaction of human with sensors changes quickly and, depending on a static model for SDC against activities, involves many challenges. In this regard, a large and growing body of literature has investigated to design a ubiquitous energy-aware SDC system for real smart home based on ML algorithms, discussed in this section as follows.

One study [7] uses triplet ML models consisting of a Naïve Bayes Classifier (NBC), a Hidden Markov Model (HMM), and a Conditional Random Field (CRF). These models are effective in average noisy data and recognize activities and compare the advanced version of this study in [15] with Support Vector Machines (SVM). SVM was later adopted for the real-world activity detection domains because it confers better results than the other conventional ML models. In an investigation [16] into Human Activity Recognition, the authors have proposed a resident Behavior Classification Model (BCM) based on supervised learning by extending the SVM, where a particular resident is identified in a group of multiple residents. The BCM evolves the sensory data and extracts distinct features from the data using morning routine data. In an analysis of Human Activity Recognition (HAR) [17,18], the authors have presented a logical interpretation of human activities. Considering HAR, research has set out to determine HAR based on cluster-based classification [11], where the authors have proposed a composite model incorporating K-Nearest Neighbor (KNN) and Dempster–Shafer theory to draw a distinction in activities instance and divide it into multiple classes inside a cluster. In [19], the authors range the current activities with the frequency of probability using the Markov logical network. In a large longitudinal study [20], two temporal models, i.e., Gaussian mixtures model and frequency map enhancement, are analyzed. The authors have investigated the previous probability frequency of activities occurring in a particular amount of time in a way to minimize the error frequency in the proposed designed algorithm. In [21], the authors overcome the data association issue, estimating the interrelationship of sensors in the context of deployment region and dimensional distance in a smart home. Furthermore, a unit graph is introduced to indicate the focus density of sensors with a colored filter to map the sensor events against the residents in a multi-residents scenario. The authors in [3] have surfaced a square box over the floorplan for the construct and have drawn the motion sensors boundaries, modeling the problems present during the tracking of many residents in the membership authorization framework. On the basis of these foundations, the researchers have begun their race to model the activities in a way to provide automated services to the users. These models detect the activity that contains embedded errors during its processing with the event-related activities [22], and the simultaneous activities made by multi-users [23,24].

Next, the research community put their full efforts to detect and recognize the activities in a dynamic environment. For instance, a resident in a smart home, performing activities of his daily routine. Data preprocessing comes in the second stage, building a foundation for the HAR preprocessing on the sensory data like event annotating, analyzing, and data labeling. The actual HAR system comes in practice by adding suitable automated tools to distinct various data for automatic tagging and monitoring objects and for the transportation of accurate learning activities to an ambient environment. In [25,26], the authors have proposed a mining-based scheme where the motion routing of the home residents is traced from the sensors data in a smart home. A number of hypotheses of the data association are carried out to the approaching sensor events with appropriate mined trajectories. The average velocity variance between multiple residents is minimized with the selection of appropriate hypotheses, and from the value of the distance and adjacency among the sensor, they calculate the average velocity variance. The scheme outperforms when the number of users is given through the mining stage. In [7], the authors use the DL model based on LSTM, which classifies and learns human activities in the smart home environment. LSTM shows a viable and significant improvement in the recognition of human activities. The comprehensive compression of the proposed scheme outperforms against the conventional DL (DNN, CNN) and ML (NB, HMM, CRF) approached using Center for Advanced Studies in Adaptive Systems (CASAS) data set. The LSTM experimental results surpass compared to the DL and ML-based schemes. The authors in [22] model the activity recognition problem by training the DNN approach based on RNN with the sensors data of smart home and predict the resident activities. To minimize the total energy usage, powerful sensors are used, which help in total energy saving. However, for filtering data, the authors use the attention module to remove meaningless information and give effective results. By applying the attention model, the LSTM and Gated Recurrent Unit (GRU) outperform in predicting the resident activities in the smart home. 

Activity prediction in smart homes is important for accurately detecting human activities to save energy consumption. Moreover, it also helps in providing the inhabitants with intelligent services and convenient daily life. In this regard, one study [27] has addressed HAR using the Artificial Neural Network (ANN) and has evaluated the precision of the proposed model by using multiple propagation techniques, i.e., Batch Back Propagation (BBP), Quick Propagation (QP), Levenberg Marquardt (LM). The author has only focused on comparing the residents’ activities. Another ANN-based scheme is proposed in [28] to model the HAR problem. In a unified framework, they train the data and extract the features using a stacked auto-encoder. In [29], the authors present an RRN-based approach for the recognition of activities in a home environment. For this purpose, various sensors are used to identify the home user. By using LSTM and GRU, they validate their proposed scheme. In [30], the authors compare different techniques to predict the approaching event of activity, the time among the current and approaching event, and the forecasting of a group of activities of an event. Moreover, the LSTM evaluation shows best predicting performance, and the other techniques are best in predicting the next activity and timestamp between the events. They express that these techniques are totally dependent on dataset selection. In another study [31], the authors use LSTM and model the HAR problem as sequences of events instead of a sequence of activities generated from a smart home data system. Firstly, the feature is extracted using LSTM and then annotated. Secondly, a standard classification method is adopted to identify the activities in the latest annotated data. They improve the annotation accuracy than the other conventional methods. In [12], the authors introduce an algorithm (Multi-Resident Tracking (sMART)), which tracks in parallel the location and the number of residents in a smart home. The proposed algorithm is tested in two data sets and compared with the two existing approaches in the state-of-art (NN-sg and GNN-sg). For the prediction of human activities in a smart home, the authors use LSTM in [32,33] and present a universal proposed solution for the activity prediction. The performance of the proposed scheme is evaluated and compared with the conventional method (Naive Bayes method). The experimental results show that the proposed scheme performs well when utilizing dense knowledge. However, this approach has suffered from the problem of overfitting due to the adaptation of a single-layered LSTM.

### 2.2. Smart Home Energy Management System (SHEMS)

Information and communication technology can be adopted in smart homes environment to improve the quality of life, especially to develop an automatic smart home energy management and intelligent control system. There are many intelligent smart approaches introduced in this research field. In [34], the authors have proposed a hybrid model for Home Energy Management Systems (HEMS) based on a prediction model using LSTM with an optimal optimization model by adopting the Genetic Algorithm (GA). The main objective of this work is to predict the power consumption and power consumption optimization in the micro-grid paradigm. Afterward, they explore the scheduling of power storage stations to stable the normal load and balance the load price by shifting the deferrable load. This balancing results in relaxing the load from the grid when using the storage station. The demand response strategy is adopted to support the automatic scheduling, and the prediction and optimization are used as integral models instead of separate models. The authors in [35] have proposed an energy management controller based on the heuristic programming approach to control the energy consumption of community buildings to reduce the cost, minimize the emission of carbon dioxide, reduce consumer discomfort level, and reduce the Peek-to-Average Ratio (PAR). Additionally, users use a renewable source of power to generate their own energy using the micro-grids station in premises. The result shows a reduction in cost, PAR, and carbon by 25.55%, 36.98%, and 24.02%, respectively, against fewer scheduled schemes.

The primary purpose of smart energy management and control system is to control energy consumption, reduce utility bills, and improve the end-user comfort level through smart techniques. To address these challenges, a novel scheme consists of three tiers based on an event optimization algorithm, as proposed in [36]. In tier one, the authors have used the historical information of each appliance form their consumption profile using clustering. Afterward, for the optimization and minimization of the computation time, in tier two, a unique event-based transition filtering and down sampling method are adopted. In the final tier, the proposed scheme uses post-processing optimization, which uses the ‘on’ and dynamic states method to enhance the accuracy of the energy of recalled appliances. The result confirms enhancement over the existing classification-based method. In reference [37], the authors have proposed a hybrid scheme incorporating an artificial neural network for the prediction model and use forthcoming hours-modified, grey wolf upgraded, differential evolution algorithm to utilize the design of an energy management controller. The prediction model forecasts the price signal using the DR strategy, and the controller schedules the household appliances against the forecasted price and energy patterns. The results show the best strategy of the proposed scheme over the existing benchmark strategy by 33.3% with regard to effective energy management. The authors have proposed a power scheduling scheme in [38] to schedule the energy consumption of community buildings regardless of non-controllable load operation time. This scheme efficiently reduces the monetary cost. Still, cost reduction is linked with the delay in the operation of appliances. 

The current research work focuses on the scheduling of high load household appliances from on-peak hours to peak hours, which leads to high discomfort level at the user end. Therefore, in [39], the authors have proposed a smart Home Energy Management System to monitor the intrusive and non-intrusive load profile, considering power consumption cost and carbon emission. The user bill is reduced with a low level of user discomfort. In [40], the authors have proposed an optimization scheme for Home Energy Management Systems based on Deep Q-Learning (DQN) and Double DQN (DDQN) to schedule the household appliances. The algorithm tremendously reduces power consumption by adopting an efficient policy in a dynamic home environment. The DDQN effectively reduces the billing cost against the DQN. The result shows that the DDQN-based scheme outperforms against Particle Swarm Optimization (PSO) approach. In [41], an automatic smart home energy management scheme is proposed based on RL to take an optimal decision during the scheduling of the appliances for the consumer. The researchers have proposed an enhanced smart home energy optimization in [42], which guarantees the controlling and scheduling of the community building environment to reduce the power consumption according to the user desire. GA and PSO algorithms are used; in contrast, power optimization and Kalman filter are adopted to vanish noise in sensor data. The result illustrates that the proposed approaches, i.e., GA and PSO, reduce the energy consumption from 27.32% to 31.42%, respectively, considering user comfort level, which is improved by 10% from 0.86 to 0.96. GA-based optimization reveals better results than PSO-based optimization. 

## 3. Problem Statement and Motivation

### 3.1. Motivation

With the emergence of the smart home paradigms, WSN has received much attention from the researchers in the past decade. Similarly, in recent years, scientists have used a variety of battery-powered low-cost ambient sensors in a smart home environment to monitor the human event of activities with environmental properties. Specifically, smart home sensors’ services may monitor the human-appliances interaction to facilitate smart home residents. Due to the high dynamic smart home environment, the smart home energy management systems continuously collect the data from sensors to avoid missing important events. However, there are a number of challenges associated with keeping the sensors active all the time, such as high energy consumption, battery depletion, etc. To cope with these issues, many conventional duty cycling schemes for WSN are introduced and keep the sensors in sleep mode when not operational. Even so, an important event can be missed when the sensor is in hibernation mode. To address energy efficiency and event detection accuracy, activity prediction-based sensor duty cycling schemes are proposed. These schemes forecast the approaching events using historical data to activate the correlated sensors. Although the predictive-based schemes suffer from a number of challenges, such as frequently missing important events, leading to system degradation. The shortcomings of the prediction-based scheme are addressed by selecting sentry-based schemes. The sentry-based schemes and the sentry sensors monitor an event of their interest when all other sensors are in hibernation mode. When a sentry sensor detects an event of interest, all other relevant sensors get activated and participate in monitoring the event. Still, localization and frequent power consumption are the major challenges in the sentry-based approach. Following is the summary of the challenges available in the current literature:

- High energy consumption and battery depletion of smart home sensors
- Inappropriate classification of sensor nodes (correlation of sentry sensor with other sensors)
- Irregular and unscheduled or unmanaged duty cycle time of sensors
- Lack of activity monitoring accuracy
- Improper location selection of sentry sensor (localization of sensor)
- Short lifetime and frequent maintenance of sentry sensor
- Lack of appropriate machine learning implementation
- Insufficient intelligence in sensors (untrained sensors)

### 3.2. Contribution

The highlights of the proposed work are as follows:

- An integrated optimized system of energy and event aware sensor duty cycling scheme has been proposed to solve the problem of ambient sensor energy depletion and detection of an expected and unexpected event in a smart home environment.
- The proposed scheme is tightly hinged with the PS, MS, and HS, which is controllable, so the sensor residual energy management and both expected and unexpected events detection are possible.
- For the detection of expected events, the BiLSTM algorithm is used, which incorporates forward and backward LSTM to efficiently predict the approaching event. The proposed scheme selects and activates the Predictive Sensors against these predicted events.
- To detect the unexpected event and minimize the sensor duty cycle rotation, the proposed scheme clusters hibernate sensor and selects MS according to their location ID, residual energy, and priority (previous frequency of event detection) using Jaccard Similarity Index (JSI).
- Finally, a practical optimization model for missing and undetected event is formulated using Q-Learning to improve the performance of the proposed scheme in terms of both expected and unexpected events detection.
- Performance evaluation results show that the proposed EEA-SDC scheme has an outclassed existing scheme in terms of sensor energy depletion, activity detection, sensor rotation, and network lifetime.

## 4. Methodology

### 4.1. Proposed Scheme Overview

The transformations of smart homes have attracted the attention of scientists to facilitate the residents through smart automation services. These smart home automation services need an automated control system using Human-Sensor Interaction (HSI) data patterns and environmental contexts. To fulfill this demand using a low-cost and battery-powered sensor, insightful research work has been carried out. Despite this interest, this research still has challenges, which need to be addressed using the proper DL algorithm. Therefore, in this work, we propose an efficient EEA-SDC consisting of two parts, i.e., (1) Event Prediction (EP) using previous history data patterns of his, (2) localization of MS to monitor the unexpected activity. EEA-SDC outperforms in event detection by manipulating the usual and unusual events. In the context of a sensor lifetime, the EEA-SDC effectively controls the energy consumption of the sensors by applying MS localization and scheduling technique.

**Figure 1:**
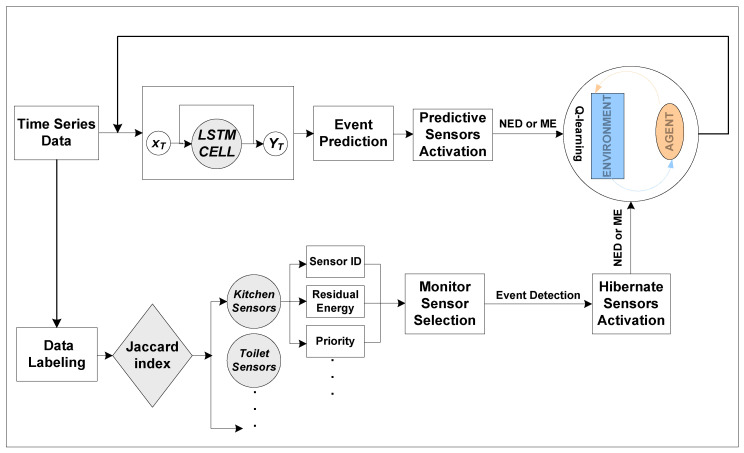
The working of the proposed EEA-SDC (Energy and Event Aware-Sensor Duty Cycling) scheme.

The main theme of EEA-SDC is to tune the sensors’ duty cycling against the event. With the aim to do so, the EEA-SDC predicts the approaching event of the smart home user using previous history data patterns. For the event prediction, the Recurrent Neural Network algorithm (RNN), i.e., BiLSTM, is used to forecast the upcoming event. Against the forecasted event, the EEA-SDC turns on the correspondent sensor and labels as PS. Thereafter, the EEA-SDC segregates the rest of the sensors into different sets of clusters on the bases of their location, residual energy, and frequency of occurrences in the previous time frame using JSI [43]. Each set of sensors represents a unique location where a user can perform distinct events and triggers a set of sensors. For instance, while watching Tele-Vision (TV) may activate sensors of drawing-room items, e.g., sofa, Air Conditioner (AC), fan, and light bulb sensors, etc.; moreover, to detect the unexpected activities, the EEA-SDC appoints and schedules an MS in each cluster of sensors based on residual energy and event detection frequency. However, there are still possibilities of missing important events; therefore, the proposed EEA-SDC scheme is optimized with a reinforcement learning algorithm called Q-Learning algorithm, and the unexpected events as a Q-Learning (QL) problem are constructed. QL addresses the problem by integrating the states, actions, and rewards. Further, the QL agents take a unique action in an environment with a high reward as compared to other actions and proceed to the next state. In this work, distinct clusters of sensors act as an environment where the high reward actions are unexpected missed events. For instance, the agent turns active a set of sensors where an unexpected activity may occur. The entire working of the proposed scheme is illustrated in Figure 1.

### 4.2. Predictive Sensors Selection

For accurate event detection in the smart home, all the sensors are divided into distinct groups on the basis of their role, i.e., PS, MS, or HS. PS group has high possibilities for detecting the event. Therefore, their sensing part is continuously functional, whereas their RF part turns on when an event occurs. Unlike PS, only the sensing part of the MS is turned on to account for the unexpected activities. On the other hand, the sensing part of the HS is turned off to save the residual energy and is unable to detect any event, but their RF part is always in low power mode.

**Figure 2:**
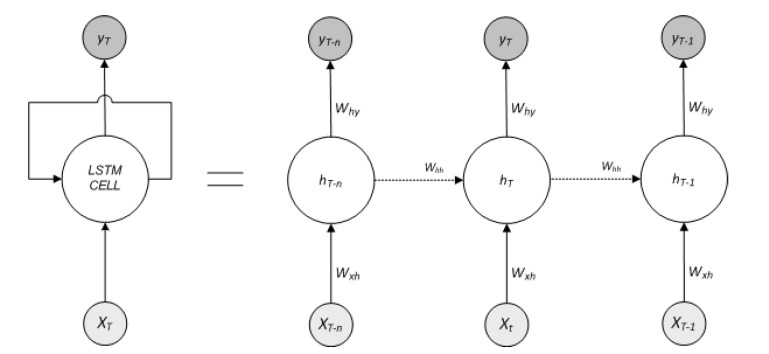
Standard unfold structure of single LSTM (Long Short-Term Memory) cell architecture working with time T steps.

**Figure 3:**
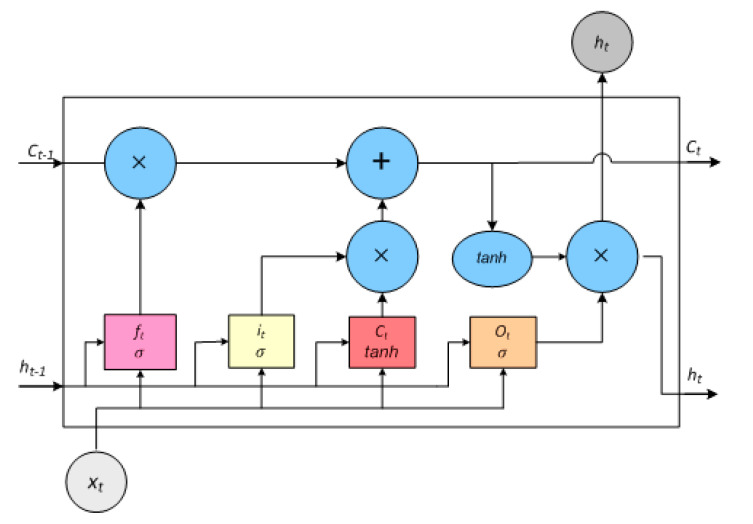
Standard LSTM Structure. The colored rectangles depict the LSTM gates, and the blue circles represent the arithmetic operation.

For the selection of PS, we use Bi-Directional Long Short-Term Memory (LSTM). The proposed approach first predicts an event of user activities, and against the predicted event, the EEA-SDC turns on the correspondent sensor to monitor the upcoming event. Further, the BiLSTM is a powerful Recurrent Neural Network (RNN) architecture, particularly designed for vanishing gradient and exploding gradient problems. Similarly, LSTM works on time sequenced-based data with long-term reliance memory. The hidden layer is the only exclusive component, which distinguishes LSTM from RNN. The hidden layer of the LSTM is known as LSTM cell, as shown in Figure 2 and Figure 3. 

The LSTM cell works by incorporating the input, forget and output gates, and a memory cell. Due to these gated networks, LSTM addresses the long-term dependency problem and grants the useful information to pass through the LSTM network. Much the same as RNN, at each time step *t*, LSTM cell processes a pair of input ‘*x_t_*’ and output *h_t_*. At the same time, while updating the parameter, the LSTM cell computes input cell state ${\tilde{c}}_{t}$ the output of cell state ‘*c_t_*’, and previous cell state output *c*_*t*−1_. Figure 3 shows the data flow in the LSTM gate structure. The current input to the cell ‘*x_t_*’ and the previous state ‘*h*_*t*−1_’ determine the output ‘*h_t_*’ and output cell state content ‘*c_t_*’ governed by the gated network of the LSTM cell. The state with the content of the cell and the output of the gates is calculated as follows:

(1) $$i_{t}=\sigma_{g}\left(W_{xi}x_{t}+W_{hi}h_{t-1}+b_{i}\right)$$

(2) $$f_{t}=\sigma_{g}\left(W_{xf}x_{t}+W_{hf}h_{t-1}+b_{f}\right)$$

(3) $$o_{t}=\sigma_{g}\left(W_{xo}x_{t}+W_{ho}h_{t-1}+b_{o}\right)$$

(4) $$\tilde{c_{t}}=tanh\left(W_{xc}x_{t}+W_{hc}h_{t-1}+b_{c}\right)$$

where ‘*W*’ is the weight matrices of the *f*, *i*, *o*, and *c* for the connection of input, output, forget gate, and cell state, respectively. ‘*x_t_*’, ‘*h_t_*’, and ‘*h_t_*_−1_’ represent the input, hidden output, previously hidden output, whereas ‘*b*’ represents the bias vectors. ‘*σ*’, ‘*g*’, and ‘*tanh*’ are the sigmoid and hyperbolic tangent activation function for the gates. On behalf of the above equations, at each iterative time ‘*t*’, out of the cell ‘*c_t_*’ and output of the layer ‘*h_t_*’, shown in Figure 3, are determined as follows:

(5) $$c_{t}=f_{t\;}\times \;c_{t-1\;}+i_{t}\times \;\;\tilde{c_{t}}$$

(6) $$h_{t}=\;o_{t}\times \;tanh\left(c_{t}\right)$$

The concluded output of the LSTM layer needs to be equal to the vector of the gross outputs and can be represented as:

(7) $$Y_{t}\;=\left[h_{T-n},\ldots ,h_{T-1}\right]$$

For instance, the prediction of an event of activities will be equal to the last component of the output $h_{T-1}$. Hence, the predicted event ‘*E*’ in the next time iteration will be $E_{T}$ = $h_{T-1}$.

**Figure 4:**
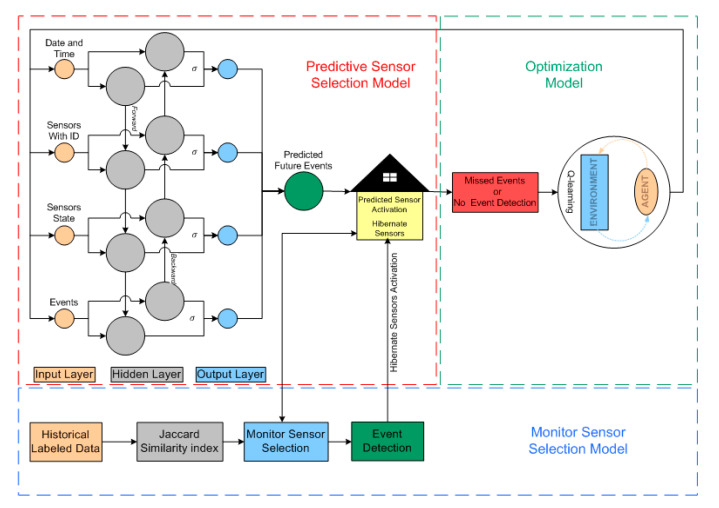
System architecture.

In this work, we adopt Bi-LSTM, which is a special type of Bidirectional RNN (BRNN), incorporating two autonomous parallel LSTM in the opposite direction, as shown in Figure 4. The unique structure of Bi-LSTM has forward and backward information log in each time step. This back and forth structure allows to execute the input in two aspects, (1) from previous information patterns to the future, (2) form future to previous patterns. Unlike unidirectional LSTM, the BiLSTM loop stores the future pattern while running backward and stores the previous and future information pattern using two hidden cells, as shown in Figure 4, to address the time dependency. The forward LSTM layer takes the input in *T* − *n* to *T* − 1 time to calculate the output $\vec{h}$, of the forward LSTM layer recursively. Unlike the forward layer, the backward LSTM layer takes the value of the reverse inputs in *T* − *n* to *T* − 1 to compute the output $\overset{\leftarrow }{h}$.

The BiLSTM produces the output *Y_T_,* where every component is computed through Equations (4) and (6).

(8) $$y_{t}=\sigma (\vec{h},\overset{\leftarrow }{h})$$

In Equation (8), σ integrates the pair of outputs; it may be a summation, multiplication, average, or concatenation function. Like pure LSTM, the BiLSTM output would be computed by Equation (7) where the final component represents the predicted event in the following time step, while assuming the event prediction.

### 4.3. Monitor Sensor Selection

The unexpected behavior of the smart home user is the major source of uncertainty in the prediction of events. Such unpredictable bizarre user events are exceptions in the user’s daily routine due to some background circumstances. For instance, a user may reach home two hours later than the routine due to a heavy traffic jam. To cope with such peculiar activities, we assign MS role amid in HS clusters and incorporate an MS’s group. During the selection of MS, we have considered issues related to missing an event and sensor residual energy. Therefore, the selection of MS incorporates three steps in a way to address the above issue accordingly. These three steps are HS clustering based on JSI using sensor location ID, assigning the role of MS to an HS using residual energy information, and sensor priority based on previous event detection history.

The JSI is a statistical method used to investigate the similarities between sets, vectors, or points. The high similarity between a pair of sets, vectors, or points indicates that there is a high resemblance, while low similarity indicates that the points are distinct. Jaccard similarity between two sets is a ratio of commonality between the two sets over all the items. If *X* and *Y* are the two sets, then JSI between these sets is computed using the ratio of the size of the intersection and the size of the union of the two sets. Or, JSI computed between two vectors or data points *X* and *Y*, where each of the vectors has a length of n, is the summation of the minimum of the two vectors divided by the summation of the maximum of the two vectors in each dimension. Practically, the numerator represents the intersection, and the denominator is the union of the two vectors. *X* and *Y* can be written as the following:

(9) $$X\;=\;\left[x_{1},\;x_{2},\;x_{3},\ldots ,x_{n}\right]$$

(10) $$Y\;=\;\left[y_{1},\;y_{2},\;y_{3},\ldots ,y_{n}\right]$$

So, the Jaccard Similarity Index of *X* and *Y* will be:

(11) $$J\left(X,Y\right)=\frac{\sum_{k=1}^{n}\min\left(x_{k}\;,\;y_{k}\right)}{\sum_{k=1}^{n}\max\left(x_{k\;},\;y_{k}\right)}$$

Or:

(12) $$J\left(X,Y\right)=\frac{\left|X\cap Y\right|}{\left|X\cup Y\right|}=\;\frac{\left|X\cap Y\right|}{\left|X|+|Y|\;-\;|X\cap Y\right|}$$

The JSI quantifies the similarity between two sensors based on three features of a sensor, i.e., location ID, value of residual energy, and previous event detection frequency. The higher the JSI value, the higher will be the similarity between the two sensors. For instance, the TV/sofa pair has a higher JSI value (JSI) than the TV/toilet pair (JSI). For MS selection, first, we annotate the sensors IDs according to their location to make clusters accordingly, such as {C1, C2, C3 … Cm}, where m represents the number of clusters created for HS. Afterward, JSI computes the sensors’ residual energy value and frequency value in each cluster’s HSC to elect the MS. By doing so, the JSI first compares the residual energy value of sensors, followed by the comparison of the previous event detection frequency value of the sensor in each cluster. The sensor event detection frequency is derived during the prediction of PS. For instance, if the user wants to prepare a meal first, he will need light in the kitchen, so for every meal, the light bulb sensor detects an event. Therefore, the kitchen light bulb sensor has a high detection frequency than other appliances in the kitchen. Finally, in each cluster Cl, every HS is taking a turn as an MS and incorporates in the MS group. The time horizon for each sensor to serve as an MS in each cluster can be calculated as Δ*T*/|*C_l_*|, where $\left|C_{l}\right|$ is the total sensor in the Cl. When MS detects an event for Cl, the role of the MS switches to PS, and the rest of the HS in the same cluster Cl also wakes up and switches to PS.

### 4.4. Proposed EEA-SDC Model Optimization

After training the model, we first validate and then test the trained model. During this phase, we investigate a couple of deficiencies, i.e., still, we are missing some events and sensor energy depletion. For instance, a resident performs an activity, but the proposed model does detect the event to cover the activity. Besides, the proposed EEA-SDC turns a sensor to PS state using LSTM-based prediction without the detection of an event. This leads to sensor residual energy depletion. To optimize our proposed EEA-SDC scheme, we optimize the performance of the proposed EEA-SDC scheme and model the problem as a Markov Decision Process (MDP). The MDP is a conventional composition of the sequential decision-making process, which works by incorporating a quantifiable set of states ‘S’, set of available actions A, state transition probability matrix P, action output in terms of Reward R, and correlation of the current and future reward discount factor γ, lying in between 0 < γ > 1. In WSN, the MDP is adopted to formulate sensor-environment interaction with the aim of sensing, activity detection, and energy optimization, etc. [44,45]. In general, MDP hinges on tuple (S, A, P, R, γ), where the agent continuously interacts with the environment and adopts an optimal policy π. The optimal policy π distribution of agent actions acquires the states:

(13) $$\pi (a|s)=\;P[A_{t}=a|S_{t}=s]$$

The grand total $G_{t}$ is the collective discounted reward in time horizon t:

(14) $$G_{t}=\;R_{t+1}+\gamma R_{t+2}+\cdots =\overset{\infty }{\underset{k=0}{\sum }}\gamma^{k}R_{t+k+1}$$

The protracted cost of the state ‘s’ determined from the cost function of the state *v_π_*(*s*) is the projected output value of the initial state ‘s’:

(15) $$v_{\pi }\left(s\right)\;={\;E}_{\pi }\left[G_{t}|S_{t}=s\right]$$

The cost function of state-action $Q_{\pi }\left(s,\;a\right)$ is determined when an action ‘a’ performs in state s and adopts the policy π:

(16) $$Q_{\pi }\left(s,\;a\right)\;={\;E}_{\pi }\left[G_{t}|S_{t}=s,{\;A}_{t}=a\right]$$

For the cost function of an action, the optimal Bellman projection equation is derived as:

(17) $$Q_{\pi }\left(s,\;a\right)=E\left[R_{t+1}+\;\gamma Q_{\pi }\left(S_{t+1},\;A_{t+1}\;\right)|S_{t}=s,{\;A}_{t}=a\;\right]$$

where $R_{t+1}$ is the reward achieved at time horizon t+1.

The optimal cost function of the state $v_{\pi }\left(s\right)$ is the highest state cost function of all the policies:

(18) $$V\times \left(s\right)={}_{\;\;\pi }^{max}v{}_{\pi }\left(s\right)$$

The optimal cost function of the action $Q_{*}\left(s,\;a\right)$ is the highest action cost function of all the policies:

(19) $$Q_{*}\left(s,\;a\right)={}_{\;\;\pi }^{max}Q{}_{*}\left(s,\;a\right)$$

The optimal action can be chosen as:

(20) $$a=\;\mathrm{argmax}Q\times \left(s_{t},a_{t}\right)$$

The optimal cost function provides the optimal result in the MDP. By knowing the optimal cost function, we can easily solve the MDP.

In many environments, the MDP is used as model-free due to the absence of a probability transition model or model transition matrix and comprehensive data demand because the transition probability matrix and reward matrix for every action are estimated on extensive data. In such environments, the cost function is optimized by the agent through episodes during the trial-error phase. Such a solution to MDP is called Reinforcement Learning (RL). In the RL method, the agent interacts with the environment and learns to figure out the best actions that accumulate maximum long-time rewards through learning or trial-error experiences. Hence, Q-Learning is one of the effective model-free RL techniques and more suitable than WSN. Moreover, the agent-environment interaction is illustrated in Figure 4, where the agent state is updated by taking action in state *s_t_*, followed by the new state $S_{t+1}$. In feedback, the agent gets a reward r$\left(s_{t},a_{t}\right)$ from the environment and iteratively updates the action-value function as follows:

(21) $$Q\left(s_{t},a_{t}\right)\leftarrow Q\left(s_{t},a_{t}\right)+\alpha [R_{t+1}+\;\gamma Q\left(s_{t+1},\;a^{\prime }\right)-Q\left(s_{t},a_{t}\right)]$$

We formulate the optimization problem as a Q-Learning problem, where ‘s’ and ‘*a*’ are the state and respective action at the sensor. The state is incorporated into the pair, consisting of Missed Event (ME) and No Event Detection (NED) due to Early-Delay Sensor Activation. While the action ‘*a*’ consists of a pair, i.e., turn to sleep (deactivation) and active mode/wake-up (activation) of the sensor. For instance, in the case of ME, if a user wants to cook a meal in the kitchen, and the cluster nominates the stove sensor as a ME. While the user wants to use only the microwave and doesn’t even touch the stove, a missing event will occur, which causes the MA. In the NED situation, the proposed scheme may activate the sensor early or delay from the happening of an event, which leads to energy depletion.

The main goal of the proposed optimization is to select an optimal policy that addresses the ME and NED to cover the energy depletion at the sensor level. We describe how the optimization solution is formulated using the Q-Learning framework as follows.

#### 4.4.1. State Space

The state space is constructed as a pair of ME and NED where:

- ME denotes the Missing Event (dependent activities)
- NED refers to the Early-Delay Activation of the sensor from the occurrences of an event

For instance, any state ‘*s_k_*’ expresses the missing activities and no activity detection in each Q-Learning episode.

#### 4.4.2. Action Space

In a particular state ‘s’, the agent can take all the available actions. In our proposed algorithm, the available actions are wake-up and sleep.

#### 4.4.3. Reward Function

When an action $a_{k}$ is taken in state ‘s’ the state responds with a value called reward value by using the reward function. The reward function cover-ups how the action is effective or poor and can be calculated as follows:

(22) $$U\left(s_{k},\;a_{k}\right)=w_{ME}U_{ME}\left(s_{k},\;a_{k}\right)+\;w_{NED}U_{NED}\left(s_{k},\;a_{k}\right)$$

where $U_{ME}\left(s_{k},\;a_{k}\right)\;\mathrm{and}\;U_{NED}\left(s_{k},\;a_{k}\right)$ are the ME and NED utility functions, respectively. $w_{ME}$ and $w_{NED}$ denote the weight factors for the Missing Event and no event detection.

## 5. Evaluation of EEA-SDC

The experimental performance evaluation of the proposed EEA-SDC scheme is comprehensively described in this section and illustrated in Figure 4. The effective simulation is carried out on the openly accessible smart home CASAS project datasets [46], i.e., Milan and Cairo. The selected CASAS datasets incorporate multi-resident data R1 and R2, where sensors are deployed in a smart home at different locations. These sensors are continuously sensing and detecting resident daily routine event activities ranging from waking in the morning till night sleeping. The detailed information of the datasets is given in Table 1, while the events tested and detected from the data sets are illustrated in Table 2, which also represents the output.

The simulation is conducted on Intel(R) Core i5 CPU with a clocking rate of 3.10 GHz, a RAM of 8 GB, and Python 3.7 in Spyder 3.3.6. The average training time per epoch is 52 s, and the total training time for all epochs is around 60 min, while the training time for both data sets is around 120 min, and the memory usage during training is around 81%.

There may be the existence of hyperparameters in the selected data, which can degrade the performance of the LSTM algorithm. To cover this loophole, the holdout method is used to optimize the hyperparameters. The model is trained on 80% data and validated and tested on the remaining 20% of the total data available in datasets. We calculate the error among the actual data and predicted data and investigate the prediction accuracy by adopting root mean squared error (RMSE) and coefficient variance of RMSE, i.e., CVRMSE. With the equation, we compute the two matrices, where *n* represents the size of the total data sample, and y, y^ show the actual and forecasted values. 

The classification model is modeled from the human-sensors interaction. Similar to our classification model, a classification model is evaluated with the Markov model and Bayesian networks-based schemes. Despite the event detection, we have compared our proposed scheme experimental results against these methods.

(23) RMSE=∑i=1n(y^i−yi)2n

(24) CVRMSE=∑i=1n(y^i−yi)2n∑i=1nyin

### 5.1. Analysis of Energy Consumption

In particular, there are three main modes, i.e., (1) Transmission, (2) Sensing, (3) Receiving, in which a sensor depletes residual energy. Especially, a sensor in MS status can only sense its surroundings for the approaching event occurrences. Therefore, MS consumes high energy on continuously sensing, before an event occurs or its status changes to PS or HS. Moreover, against the event detection, the status of MS and the approaching event to the same MS group sensors alter the mode to PS and start consuming energy upon receiving and transmitting the information. Additionally, the sensor energy depletion is directly proportional to the resident’s event of activities and the number of the residents; and more frequent activities and more residents result in high energy consumption. The overall energy consumption of the smart home network is evaluated by employing the energy model presented in [47].

**Figure 5:**
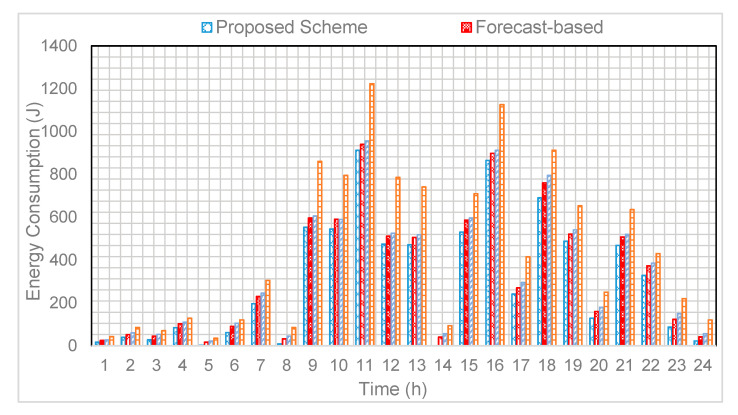
Energy consumption of sensors compared to relay and forecast-based schemes.

**Figure 6:**
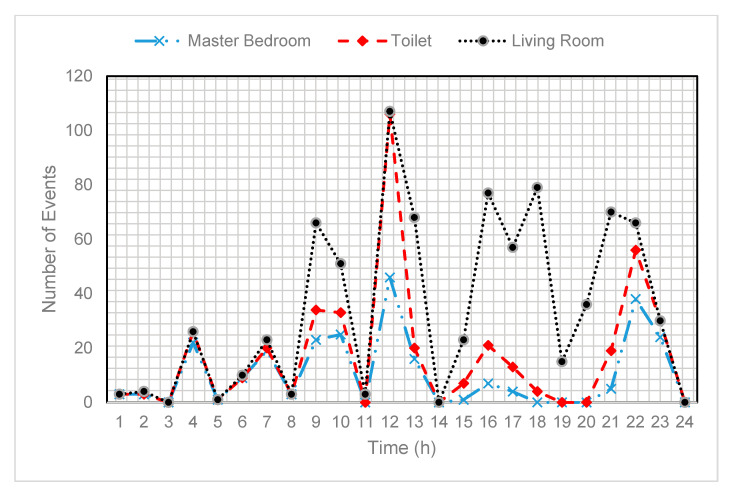
Several activities performed by the smart home resident in the master bedroom, toilet, and living room.

As discussed earlier, the sensor energy depletion is dependent on the number of residents and associated events, i.e., more residents exhibit frequent event of activities, which leads to high energy consumption. The Cairo data set consists of two users; therefore, the probability of event detection is high, and the sensor activation is more frequent as compared to a single resident dataset. The energy consumption comparison of the proposed scheme EEA-SDC against the forecast and relay-based approaches is shown in Figure 5. The purpose of illustrating these comparative results is to endorse that the BiLSTM is the appropriate choice for the training and prediction. At the time of experimental evaluation, we have observed that the forecast and relay-based approaches activate various sensors inaccurately and lose important events of activities. Hence, these problems degrade the performance of the forecast and relay-based approaches. In Figure 6, we identify a specific activity of the resident in different time slots of a day. This analysis shows that the long-term data prediction can be utilized for finding the time sequence information of the resident while performing a specific activity. Therefore, this information can be utilized to determine the particular sensors of whom residual energy will be drained quickly. During the 24 h of time, we have noticed that during the daytime, the energy consumption of some sensors, such as a morning and evening time in the kitchen and toilet, is high compared to other times of the day. It also depends on the nature of the dataset; for instance, the users in both datasets remain at home instead of going outside. Therefore, we can see the energy consumption of the sensors is high in the daytime.

The network lifetime of the EEA-SDC is compared with the forecast and relay-based approaches, and their results are shown in Table 2; we observe that the accuracy of EEA-SDC event detection is high, which minimizes the extra energy-draining of the sensor and significantly prolong the battery lifetime of the sensors. Besides, we observe that the proposed EEA-SDC scheme swaps the MS; the swapping of MS additionally improves the battery lifetime of the sensor in the context of energy consumption. The relay-based approach continuously elects sensors for the event detection; such continuous election of sensors leads to the high energy consumption of the sensor and concurrently degrades the network lifetime, as depicted in Table 3. The prediction-based approach activates a sensor against unsuitable time without using the previous knowledge. Hence, the prediction-based approach frequently activates sensors in inappropriate time (no activity or missed activity) and continuously misses events along with the high energy consumption and also curtails the network lifetime.

### 5.2. Analysis of Detection Accuracy

**Figure 7:**
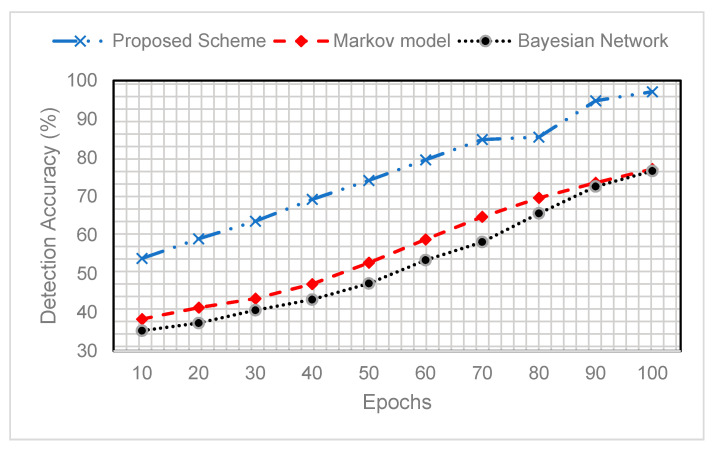
The accuracy of the event detection compared to the Markov model and Bayesian network.

In this sub-section, we evaluate the accuracy of the event detection of the proposed EEA-SDC scheme with the Markov model and Bayesian network-based schemes. Figure 7 shows that the accuracy of the event detection of the proposed scheme is tremendously high against the conventional ML algorithms. The initial training episodes show that the accuracy of the event detection is low; later, adding more number of episodes exhibits high accuracy of the event detection. Moreover, the optimization of our proposed scheme through the Q-Learning algorithm is to acknowledge those events which are not detected during the training and testing of LSTM or missed during prediction. Hence, the experimental result shows that the Q-Learning algorithm phase improves the event detection with the assistance of awarding the missed or not detected events negatively, withdrawing for the next episode. Eventually, we also observe that composite BiLSTM with the Q-Learning surpasses the ML algorithms due to incorporating forward and backward long-term memory and necessarily extracts the future and past spatiotemporal information. Therefore, the BiLSTM minimizes the preprocessing time and improves the feature extraction using past and future information. Table 4 represents the comparative study of the proposed with the conventional ML algorithms in the context of the accuracy of the event detection.

**Figure 8:**
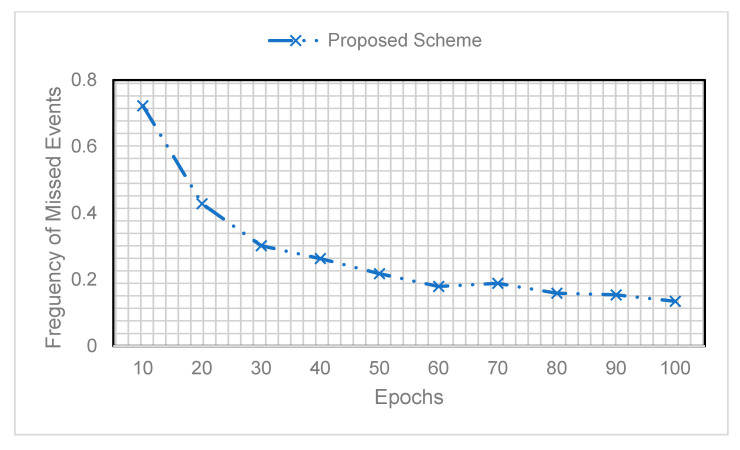
Frequency of the missed events.

As we can see, the proposed EEA-SDC performs better in achieving significantly high detection accuracy compared to the Markov and Bayesian models. The Bayesian and Markov result in minimum detection accuracy because the former works on the conditional probabilities, and the latter works over states. Both of them have limitations, such as Markov performs lower in the case of unlabeled data because it relays on the present state of the information. Besides, the Bayesian networks work better whenever the dependency of the events on each other is clearly graphed as directed acyclic graphs. Therefore, the main idea of the Bayesian networks is to find the conditional probabilities of an event A, given the probability of event B. Interested readers can refer to [48]. Figure 8 shows the frequency of the missed events when validating our model. It is noted that the frequency of missing events in the initial episodes is very high. Afterward, it is noticed that with the greater number of epochs, the frequency of missed events is significantly decreased. The reason for a smaller number of missing events in the final episodes is the parallel execution of BiLSTM and Q-Learning algorithms during the training process. The minimum number of missing events significantly increases detection accuracy.

(25) $$P\left(A|B\right)=\;\frac{P\left(B|A\right)P\left(A\right)}{P\left(B\right)}$$

## 6. Conclusions

In this research work, we propose an EEA-SDC scheme based on the composite structure of BiLSTM with the Q-Learning technique. The proposed scheme models the smart home resident’s event of activities as a time sequence information problem. Similarly, the proposed scheme predicts the approaching events of the resident, a smart home user. Further, against these predicted events, particular sensors are activated in order to record the event of activities. However, the unusual events are monitored using a cluster of the Hibernate Sensor with an elected cluster head named Monitor Sensor. The MS is elected on the basis of location ID, residual energy, and previous frequency of occurrences. Finally, we compare the performance of our proposed scheme with the existence of sensor duty cycling schemes based on conventional ML algorithms. Eventually, the proposed scheme exhibits the detection accuracy of 96.12% using the data set with more than one resident. The accuracy reveals the energy consumption of the designated sensors, and the overall network lifetime is remarkably enhanced against the ML-based schemes.

In the future, we are planning to extend this research work to other fields, such as smart cities and smart grid energy management. A network of interconnected systems will be formed where the agent of a home within a smart city will be communicating in parallel with another home and smart grid.

## Figures and Tables

**Table 1:** CASAS datasets information.

Datasets	Milan	Cairo
No. of sensors	28	27
Label of sensors	Motion(M), Door (D), Temperature (T)	M,T,
Total no. of residents	1 + pet	2 + pet
Total no. of events	15	13

**Table 2:** The tested and detected events in CASAS datasets.

Events	Milan	Cairo	Detection Accuracy (24 h)
Cooking	Kitchen activity Dining room activity	Breakfast Dinner Lunch	100%
Snoozing	Sleep	Resident 1 (R1) sleep Resident 2 (R2) sleep	100%
Toileting	Bed to toilet	Bed to toilet	75%
Working	Chores Desk activity	R1 work in an office Laundry	100%
Waking up	Morning meds	R2 wake R1 wake	100%
Eat	Kitchen activity Dining room activity	Breakfast Dinner Lunch	100%
Leaving home	Leave home	Leave home	50%
Relax	Watch TV Reading	Night wandering	75%
Medication	Morning meds Evening meds	R2 take medicine	100%
Showering	Master bathroom Guest bathroom	-	25%
Others	Master bedroom activity	-	100%

**Table 3:** Network lifetime hypothesis compared to relay and forecast-based schemes sensors.

Scheme	Network Lifetime
EEA-SDC	137
Rely-based	78
Forecast-based	104

**Table 4:** The accuracy of the event detection of the proposed EEA-SDC with the Markov model and Bayesian network in the case of the Milan dataset.

Algorithms	Event Detection Accuracy
EEA-SDC	97.12%
Markov model (Scheme in [32])	77.1%
Bayesian network (Scheme in [32])	76.62%
